# Quasi-static testing of UHPC cupped socket piers-footing connection and its seismic fragility analysis under near-fault ground motions

**DOI:** 10.1038/s41598-024-58543-4

**Published:** 2024-05-13

**Authors:** Dahai Yang, Zhigang Wu, Rui Zuo, Jianluan Li, Haihui Xie, Yingao Zhang

**Affiliations:** 1 National Key Laboratory of Bridge Safety and Resilience, Beijing University of Technology, Beijing, 100124 People’s Republic of China; 2 Anhui Transport Consulting and Design Institute Co., Ltd, Hefei, 230088 Anhui People’s Republic of China; 3 Department of Civil Engineering, Hefei University of Technology, Hefei, 230009 Anhui People’s Republic of China

**Keywords:** Assembly construction, UHPC, Piers-footing connection, Quasi-static testing, Seismic fragility analysis, Civil engineering, Energy infrastructure

## Abstract

Assembly construction is extensively employed in bridge construction due to its ability to accelerate construction and improve quality. To speed the recovery of bridges after major earthquakes, this study proposes an assembled connection for precast piers and footings based on assembly construction. The precast piers are connected to the footings using ultra-high-performance concrete (UHPC) post-cast cupped sockets. Two specimens are tested with a 1:4 scale, namely, the cast-in-place (CIP) specimen and, the UHPC cupped socket pier specimen. Finite element models (FEM) of a continuous girder bridge with cupped socket connections are developed and verified by experimental results. The seismic fragility analysis is conducted to investigate the difference between the cupped socket connection and the CIP connection. The experimental results showed that the plastic hinge was formed on the precast piers and there was little damage to the UHPC sockets. The results of FEA indicate that UHPC cupped socket piers have slightly higher seismic fragility than the seismic fragility of cast-in-place piers. Then, some methods were proposed to reduce the seismic fragility of UHPC cupped socket piers, and their availability was confirmed by comparing them with the seismic fragility of CIP piers. Finally, an example bridge with this connection is introduced to illustrate replacing prefabricated piers after an earthquake.

## Introduction

The prefabricated assembly stands out among the emergent Accelerated Bridge Construction (ABC) due to its capacity to significantly reduce construction time and improve the quality of the constructed structure. This approach is frequently used in offshore, densely populated urban areas, and environmentally challenging locations^1,2^. However, while bridge superstructure assembly technology is rapidly developing and widely used in actual engineering, there are fewer instances of substructure assembly using prefabricated components, particularly in medium and high seismic regions^3^. The sufficient connection strength between precast members under earthquake is a key constraint in the assembled construction of substructures^4^. Currently, there are more connection methods available for bridge piers and footings that can achieve similar seismic performance as CIP piers. However, these methods are also prone to causing damage during earthquakes. Billah^4^ and Capani^5^ proposed the use of new materials, such as carbon fiber reinforced polymer (CFRP) and engineered cementitious composite (ECC), to retrofit damaged bridge piers. However, this approach is only suitable for temporary bridge applications. Replacing damaged piers to ensure the long-term usability of a bridge has become a popular research topic among scholars^6,7^.

The main types of bridge piers-footings connections include post-tensioned tendon connections, grouted connections, and socket connections^8^. Post-tensioned reinforcement connections are frequently used in precast segmental construction^9^. Sideris et al.^10^ investigated segmental bridges using shake table tests and quasi-static tests and concluded that segmental bridges showed high ductility under severe earthquakes. White et al.^11^ investigated the seismic performance of post-tensioned non-emulative column-footing connections and confirmed that the use of this connection develops the seismic performance of piers. However, this connection leads to plastic hinges not only at the piers but also at the pier-footing interface, As a result, the superstructure experiences large deformations due to the swaying of the piers^12^. Grouted connections are made by inserting a prefabricated column into a recess reserved for the cover beam or foundation and then filling it with grout. The safety of this connection has been studied and proven^13–15^, but the compactness of grouting in narrow space is difficult to detect, which affects the use of this connection in strong earthquake areas. Currently, there is another method of grouting the connection by flowing out a certain length of the hollow area at the end of the abutment and grouting into the hollow area after placement onto the cover beam or foundation^16,17^. This connection is easier to grout and can sustain a similar state of damage as cast-in-place piers. However, the reinforcement through the foundations and piers makes it impossible to replace only the piers after an earthquake^18^.

The conventional socket connection is generally slotted on the footing, then the precast pier is inserted and grouted^19,20^. Zhang et al.^21^ investigated the design parameters of individual piers using this connection, but they did not examine the seismic performance of this connection in an actual bridge. Haraldsson et al.^22^ tested the socket piers through lateral-load tests, which showed that the seismic performance of the pier using the connection is as good as that of a comparable CIP pier. It’s noted there is no reinforcement between the bridge piers and the footings with this socket connection. Although this feature facilitates the replacement of the bridge pier after an earthquake, slotting in the footings will compromise the integrity of the footings.

This study proposes a new UHPC cupped socket to connect the precast pier and footing. UHPC has higher strength and better performance under various loads^23,24^. The pre-constructed cupped sockets are poured on the bridge footings, allowing for the direct placement and pouring of the precast piers into the sockets. Initial estimates showed that constructing an actual bridge using this connection could save time and accelerate the construction speed. In comparison to conventional socket connections, this method prevents the need for cutting holes in the footings and truncating the reinforcement, thereby ensuring the integrity of the footing. Additionally, it facilitates the swift restoration of bridge functions after major earthquakes by replacing damaged piers easily. The seismic performance of the cupped socket connection and CIP connection piers is compared by the quasi-static tests. Finite element models of a continuous girder bridge using this connection are developed to analyze the fragility of the piers and investigate the seismic performance of the connection. Finally, a post-earthquake replacement instruction is proposed based on the features of this connection to complete the rapid replacement of damaged bridge piers after the earthquakes.

## Experimental research

### Concept description

**Figure 1:**
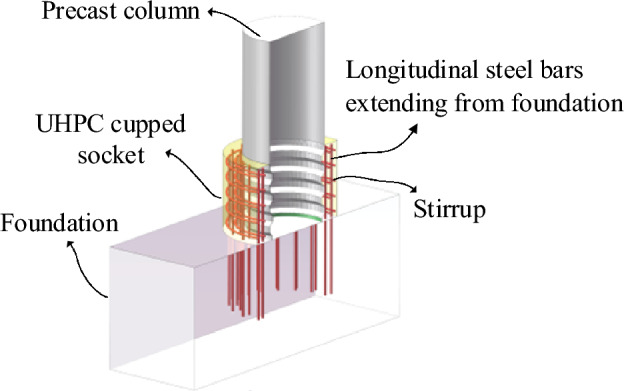
The schematic diagram of the socket connection.

As shown in Fig. 1, the UHPC cupped socket connection includes a precast column, two rings of prominent longitudinal reinforcement from the footing, and a certain thickness of concrete socket around the piers. There are no rebars across the column and footing, and this detail makes the vertical load directly transferred to the footing through the roughened interface, which facilitates individual replacement of members.

This test is based on the background of the actual project in northwest China. Due to the impact of the natural environment, the construction period of the engineering project is short. Therefore, assembly construction was necessary to shorten the construction time. The seismic performance of a structure using socket connections is primarily determined by the piers’ embedded depth and the lateral constraint^15^. The embedding depth of the column is 0.9 times the diameter of the column as suggested by existing studies^3^, so 0.9D embedding depth is used in the actual bridge.

**Figure 2:**
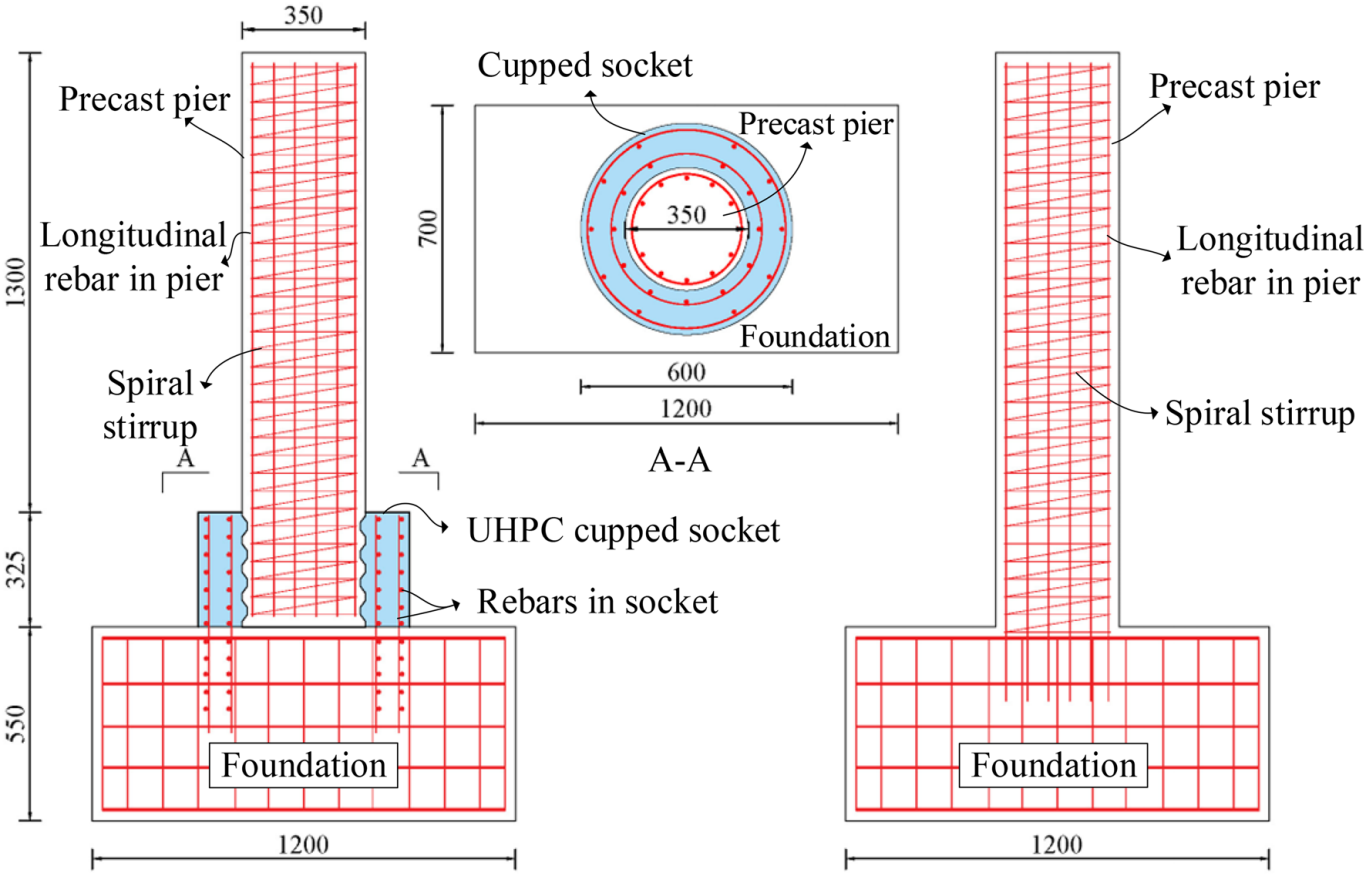
The details of the two specimens (unit: mm).

The size of the test specimens is scaled from the actual bridge piers. The height of the actual piers’ cupped socket is 1300 m and the thickness of the actual piers’ cupped socket is 500 mm. The scaling ratio for this test was 1:4, so the height of the cupped socket of the test specimen was 325 mm and the thickness was 125 mm. Figure 2 displays the specimen size and the steel bar layout.

The footing measures 1200 mm in length and 550 mm in height. Two rings of 12 mm diameter longitudinal rebars are extended uniformly to connect the foundation and the cupped socket, and the longitudinal rebars are surrounded by 8 mm diameter stirrups. The spacing of spiral stirrups in precast piers and concrete sockets is both 5 mm. The height of the cupped socket is 325 mm and its thickness is 125 mm. The parameters of the three groups of test components are shown in Table 1.

**Table 1:** Detailed parameters of the specimens.

Specimen ID	Height of column (mm)	Diameter of column (mm)	Height of socket (mm)	Thickness of socket (mm)	Ratio of reinforcement (%)	Socket material
CIP	1625	350	N.A	N.A	1.36	N.A
UC-1	1625	350	325	125	1.36	UHPC

**Figure 3:**
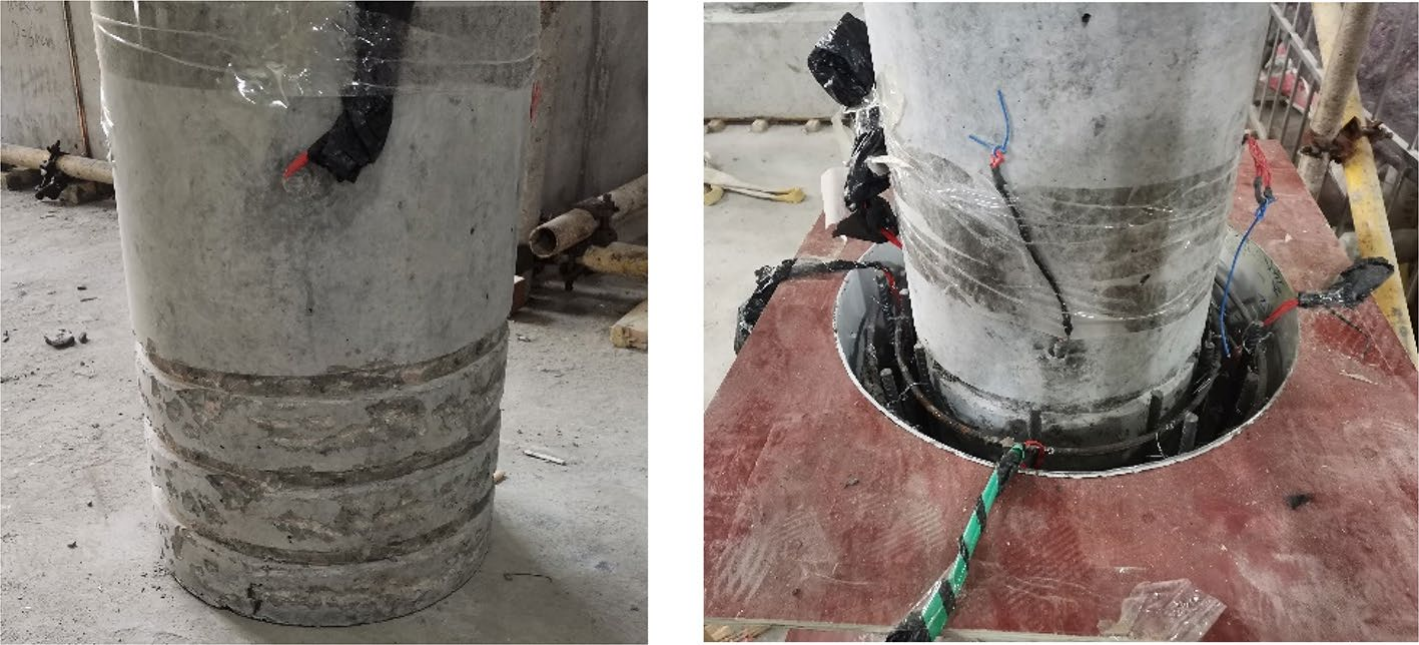
The grooved surface at the bottom of the column.

The column and footing are prefabricated simultaneously in the factory. Once the prefabricated specimen is maintained, the precast specimens are poured and connected in the same factory. It’s noted that before pouring the sockets, the bottom of the column surfaces are grooved to increase the shear capacity between columns and sockets, as shown in Fig. 3.

### Material test

**Figure 4:**
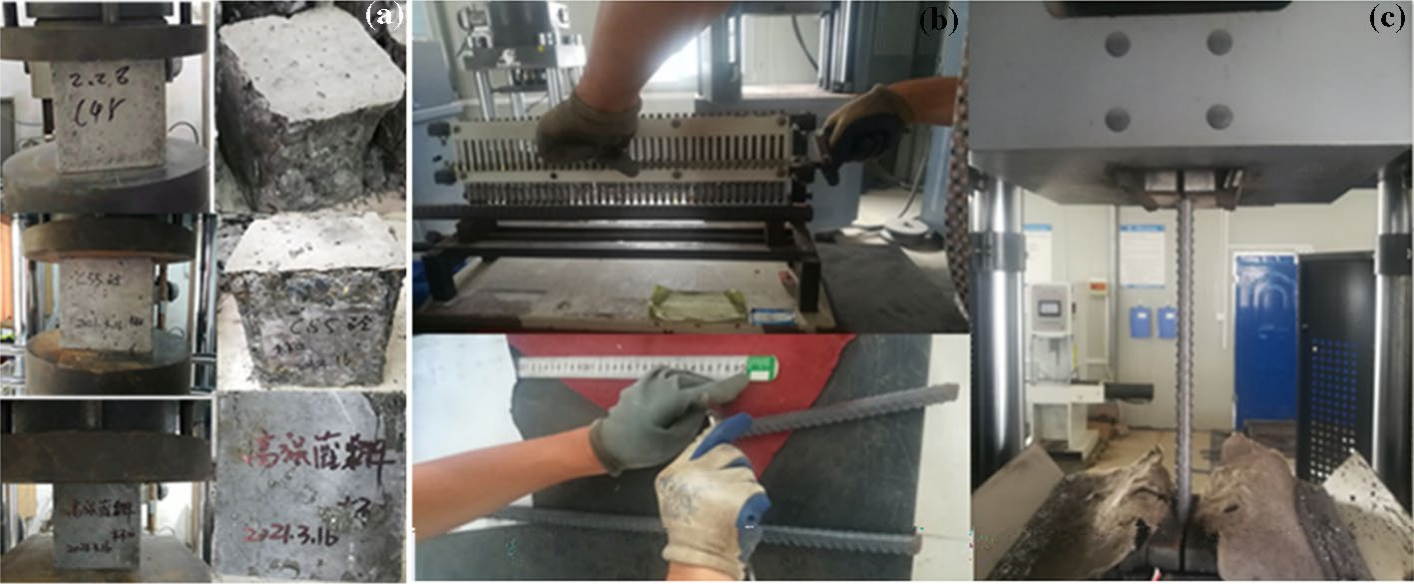
(**a**) Compressive test of concrete; (**b**) tensile test of compression test.

The study utilized C40 and C55 concrete, as well as U120 high-strength concrete, by the Chinese code for the design of concrete structures GB 50010-2010^25^. The strength of stirrups and longitudinal reinforcement is HPB300 and HRB400, respectively. Material tests were conducted following the Chinese standard^26,27^, and the compressive strength of ultra-high strength concrete and common concrete is shown in Table 2. The material tests are shown in Fig. 4, and Table 3 displays the tensile strength of the rebars.

**Table 2:** Strength of UHPC and concrete.

Element	Material	Specimens	Measured properties (MPa)	Elastic modulus (Mpa)
Precast column/footings	C40 concrete	Cubes (150 mm)	42.3	33,000
Cuppedsocket	UHPC	Cubes (150 mm)	128.5	41,200

**Table 3:** Tensile strength test of reinforcements.

Material type	Yield strength *f*_*y*_ (Mpa)	Ultimate strength *f*_*u*_ (Mpa)	Elongation (%)
HPB300 (D = 8 mm)	325	440	27
HRB400 (D = 12 mm)	420	616	18

### Loading setups, and loading procedure

**Figure 5:**
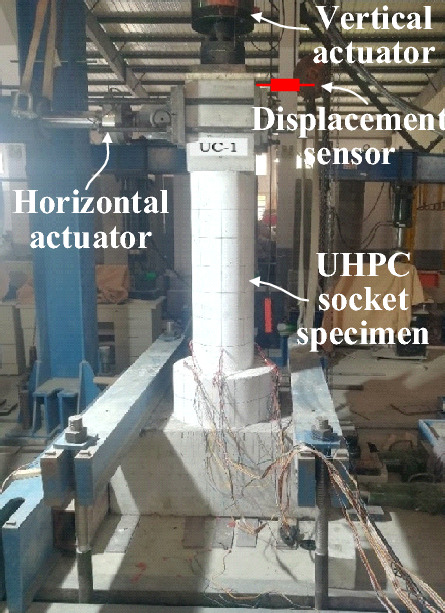
Test setup.

**Figure 6:**
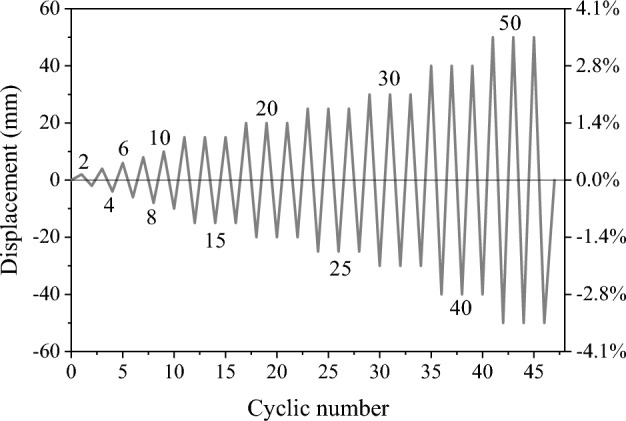
Loading pattern.

The test setup (Fig. 5) consists of a horizontal actuator and a vertical actuator. The measuring range of the vertical actuator is 1000 kN, and the maximum stroke is 1000 mm. The measuring range of the vertical actuator is 500 kN, and the maximum stroke is 500 mm. The horizontal actuator's center is 1500 mm away from the specimen's bottom, and the displacement sensor is installed at the specimen's top, approximately 1600 mm from its bottom. The designed axial load is 427 kN, and the force of the brake is monitored by the oil pressure chamber. The horizontal load is controlled by displacement, and the cyclic load application method is shown in Fig. 6.

### Damage processes

**Figure 7:**
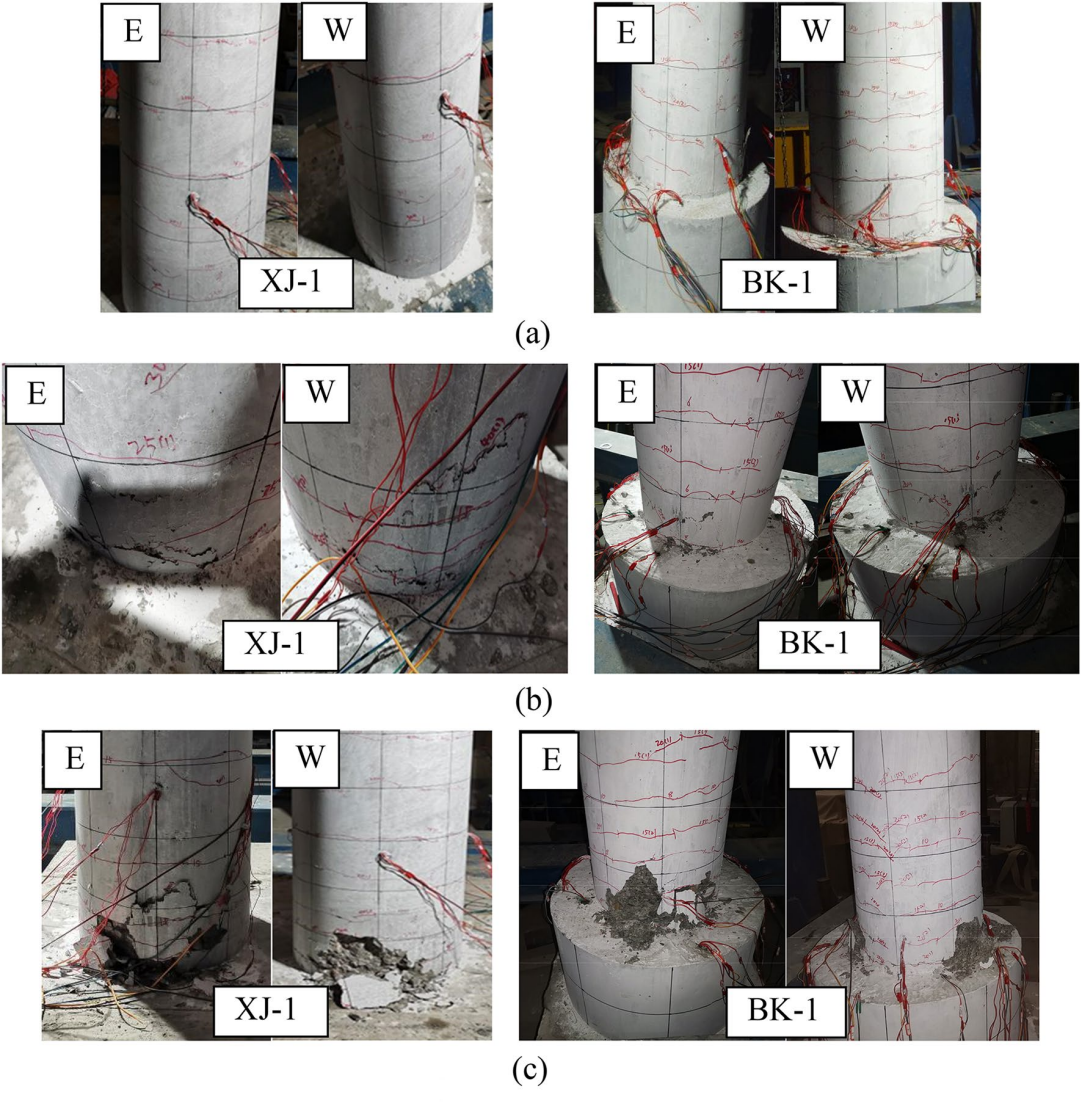
Damage development and final failure state of the specimens. (**a**) Fully developed cracks. (**b**) Concrete spalling. (**c**) Final failures state.

The damage development and the final failure state of the two specimens were the same, as shown in Fig. 7. Horizontal cracks began to appear in the cast-in-place column at 35 mm from the column bottom when the drift ratio reached 0.62%. As the drift ratio increased to 1.85%, the cracks developed maturely and ran through in the loading direction. The concrete spalls within a height of 150 mm from the bottom of the column when the drift ratio reaches 2.46%, and finally, the concrete at the bottom of the column is crushed when the drift ratio reaches 3.07%. Cracks start to appear in the socket-connected columns when the drift ratio reaches 0.46%, which is earlier compared to the CIP columns. At the drift ratio of 1.92%, cracks develop sufficiently but are relatively dense. When the drift ratio reached 3.07%, the concrete above the contact surface of the column was similarly crushed.

**Figure 8:**
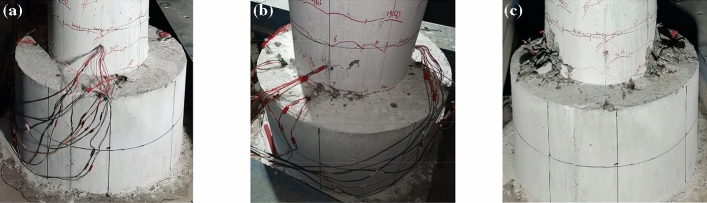
The state of the UHPC socket throughout the test. (**a**) Fully developed cracks. (**b**) Concrete spalling. (**c**) Final failures state.

Throughout the test, the UHPC sockets remained intact even when the piers were damaged, as shown in Fig. 8. This indicates that the sockets can be reused when the precast piers are replaced after major earthquakes.

### Force–displacement curves

**Figure 9:**
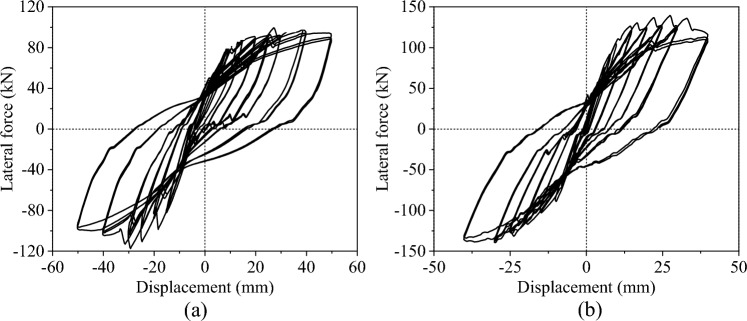
Hysteretic response of the specimens. (**a**) CIP. (**b**) UC-1.

Figure 9 shows the hysteretic curves of the two specimens. Comparing the hysteretic curves, the lateral force of the CIP pier is lower than that of the cupped socket pier. The major reason is that the UHPC socket has a stronger constraint to the column, which improves the stiffness of the column, leading to a lower effective height of the socket pier. However, the increase in column stiffness reduces its ductility, which will be discussed in detail in the fragility analysis later on.

### Strain of longitudinal rebars

**Figure 10:**
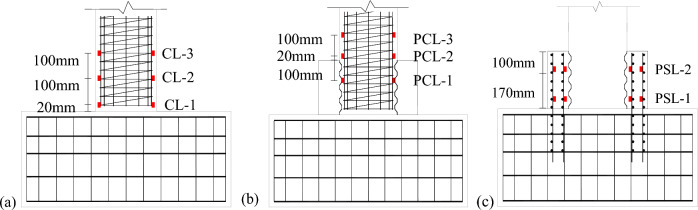
Locations of strain gauges: (**a**) CIP; (**b**) longitudinal reinforcement of column of UC-1 and (**c**) longitudinal reinforcement of cupped socket of UC-1.

Figure 10 shows the placement of the rebar strain gauges in the two specimens. These strain gauges are used to measure the strain of the longitudinal reinforcement in the columns and socket.

**Figure 11:**
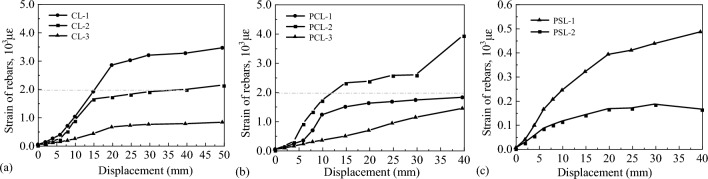
Strain responses of longitudinal reinforcement at different locations.

Figure 11 shows the strains of reinforcement at different displacements. Figure 11a illustrates that the plastic hinge of the CIP specimen occurs at the bottom of the column, where the peak reinforcement strain is close to 0.004. Figure 11b,c demonstrate that the plastic hinge of the UC-1 specimen occurs at the column above the cupped socket and the maximum value of reinforcement strain inside the cupped socket is 0.0005, which is significantly below the yield state.

## Bridge prototype and numerical model

### Bridge prototype

**Figure 12:**
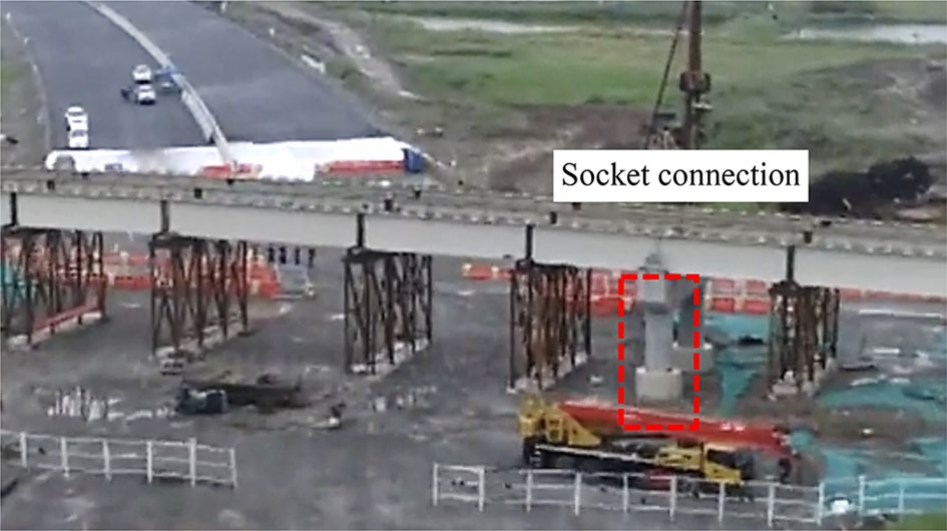
Application in a bridge in northwest China.

The prototype bridge studied in this paper is a small-span steel–concrete beam in northwest China, as shown in Fig. 12. The superstructure consists of three equal-section I-beams with a span of 45 m, connected by small crossbeams arranged in the cross-bridge direction. And 25 cm precast concrete slabs are erected on the I-beams. The main span piers are connected by sockets, with a height of 1.3 m.

Ultra-high-performance concrete (UHPC) is preferred for cupped sockets due to its rapid strength gain and short curing period. However, if the construction period allows, common concrete can be used to reduce costs. To investigate the impact of different socket materials on the cupped socket connection, the finite element analysis was conducted using a specimen of the same size as UC-1, but with C50 concrete as the socket material. The seismic performance of piers connected by the CIP method, cupped sockets using C50 concrete, and sockets using UHPC are analyzed separately to compare the impacts of the cupped socket connection and the CIP connection. The details of the three types of piers are shown in Table 4.

**Table 4:** Details of columns.

Element	Height of socket	Diameter of socket	Height of column	Diameter of socket	Material of socket concrete	Material of column
Precast column/Pile cap (CIP)	–	–	6.5 m	1.4 m	–	C40
Cupped concrete socket (UC-1)	1.3 m	2.4 m	5.2 m	2.4 m	C50	C40
Cupped concrete socket (UC-2)	1.3 m	2.4 m	5.2 m	2.4 m	UHPC	C40

### Development and verification of finite element models

**Figure 13:**
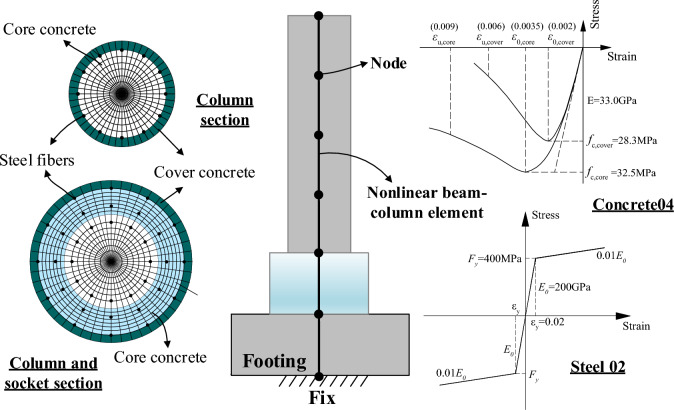
Finite-element models of the single pier in OpenSees.

Finite element models of single piers have been developed using OpenSees^28^. The links between the piers and sockets are bound with a fix at the bottom of the footings. Fiber sections of columns and sockets are developed according to the instructions in Fig. 13. It also illustrates the concrete and reinforcement stress–strain models and provides specific parameters for C40 concrete and HRB400 reinforcement. The mass of the model is distributed over the nodes and the elements between nodes are nonlinear beam-column elements.

**Figure 14:**
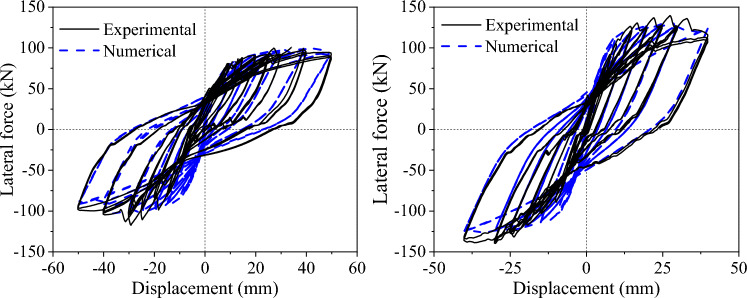
Comparison of experimental and numerical hysteretic responses.

Figure 14 compares the hysteresis curves obtained from the simulation with those obtained from the quasi-static tests. In the experimental results, the initial stiffness of specimens is lower because the relative displacement between the actuator and the strongly reactive wall slightly reduces the initial stiffness. After calculation, the numerical results of the yield force of the CIP, and UC-1 differed from the test results by 7.1% and 1.8%, respectively, and the peak forces differed by 1.5%, and 7.5%, respectively, which indicated that the FEM of piers is reasonable.

**Figure 15:**
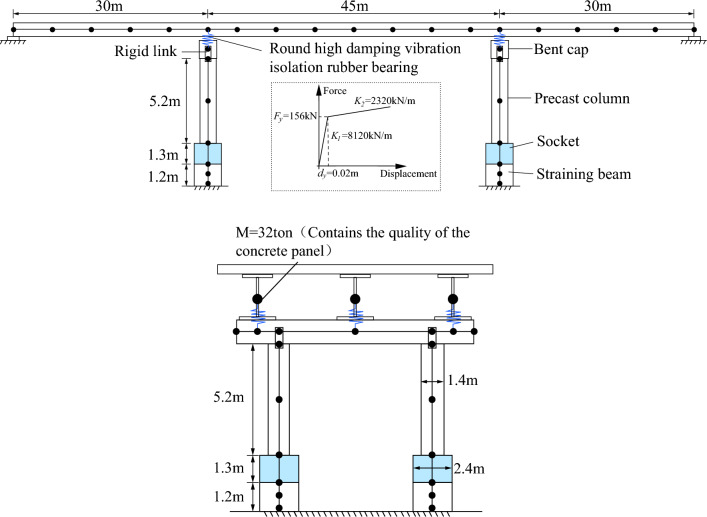
Prototype bridge and finite element modeling in OpenSees.

Then, the real bridge model (Fig. 15) is developed by using the verified material property parameters and modeling method of the single-pier model. The mass of the bridge is assigned to the nodes. The girders are not the focus of this study and generally sustain less damage under earthquakes. So they are developed using linear-elastic elements. This numerical model will be used for the following fragility analysis.

## Seismic fragility analysis

### Fragility function

Seismic fragility gives the probability that a structure will occur or exceed a specific damage state at a specified ground shaking intensity, facilitating the evaluation of bridge seismic performance^29–32^. The conditional probability is given by a general equation:

1 $$Fragility=P[D\ge C|IM]$$

where *IM* is the abbreviation of intensity measure; *D* is the structure response; and *C* is the capacity of the specified damage state.

To use Eq. (1) for fragility evaluation, a probabilistic seismic demand model (PSDM) conditional on *IM* is established, and PSDM provides the correlation between engineering demand parameters (e.g. drift ratio, curvature) and earthquake intensity measure (e.g. peak ground acceleration (PGA), peak ground velocity (PGV)) through the following equation:

2 $${\text{ln}}\left(EDP\right)={\text{ln}}\left(a\right)+b{\text{ln}}(IM)$$

where both *a* and *b* are regression coefficients. Furthermore, the distribution of the demand about its median is often assumed to follow a two-parameter lognormal probability distribution. And the dispersion (${\beta }_{EDP|IM}$) of the demand, which is conditioned on the *IM*, is calculated using Eq. (4)

3 $${\beta }_{EDP|IM}=\sqrt{\frac{\sum_{i=1}^{n}{[{\text{ln}}({EDP}_{i}-{\text{ln}}(a{IM}^{b})]}^{2}}{n-2}}$$

where *EDP*_*i*_ and *aIM*^*b*^ are the calculated seismic demands from the dynamic analyses and the PSDMs, respectively, and n is the number of dynamic simulations. Many scholars^33–36^ assumed that the distributions of the component capacities were assumed to be lognormal. Therefore, the conditional probability of exceeding a specified damage state for a given IM can be calculated using Eq. (5)

4 $$P\left[D\ge C|IM\right]=\varnothing (\frac{{\text{ln}}(a{IM}^{b})}{\sqrt{{{\beta }^{2}}_{EDP|IM}+{\beta }_{c}^{2}}})$$

where $\varnothing ( )$ is standard normal cumulative distribution function; and ${\beta }_{c}$ is the dispersion of the capacity.

### Definition of damage states

The damage index is a quantification of the structural capacity and is a prerequisite for fragility analysis^37,38^. Curvature ductility of piers (${\mu }_{\varnothing }=\varnothing /{\varnothing }_{y}$, where $\varnothing$ and ${\varnothing }_{y}$ are the curvature demand and yield curvature, respectively) is often used in the study of bridge fragility, so this parameter is employed. The definition of the different damage states was based on the previous study by Wei^39^. Curvature ductility of the bridge piers in this study for the four states was determined through moment–curvature analysis and the results are shown in Table 5.

**Table 5:** Definition of different damage states.

Damage state (DS)	Description	Corresponding concrete strain	Curvature ductility
Slight damage (DS1)	Cracks on the concrete surface	0.0006	0.8
Moderate damage (DS2)	Slight spalling of the cover concrete	0.0020	3.1
Extensive damage (DS3)	Extensive spalling of the cover concrete	0.0035	6.3
Complete damage (DS4)	Complete spalling of the cover concrete in the whole plastic hinge	0.0060	10.6

### Earthquake selection

The uncertainty of the structural demand in the fragility analysis comes from a large number of non-linear time history analyses^40^. Selecting ground motions that match the design response spectrum of the site is the ideal approach. However, the basic seismic intensity of the case study bridge site was VI and no strong motion records had been detected. Therefore, 20 actual near-fault records from the PEER ground motion database were applied as the ground motion input instead of the specific ground motion of the bridge site, which to a certain extent made the research results more widely applicable, as shown in Table 6. The selection criteria for these ground motions is that they must be within 10 km of the fault. This results in more energetic shocks that tend to cause greater damage to buildings. To obtain more results from the non-linear time history analysis, the original 20 ground motions were gradually linearly reduced and enlarged to form more ground motions^41^. In addition, this linear adjustment (after scaling using 6 scale factors 0.5, 1.0, 1.5, 2.0, 2.5, 3.0) resulted in a wider range of ground motion intensities, with the PGAs and PGVs range becoming 0.06–2.55 g and 3.5–400.2 cm/s after scaling.

**Table 6:** Summary of selected ground motions.

No	Earthquake	Year	Station	Magnitude	Distance to fault (km)	PGA (g)	PGV (cm/s)
1	Imperial Valley	1940	El Centro Array #9	7	6.1	0.28	30.9
2	Imperial Valley	1979	El Centro Array #5	6.5	4	0.53	48.9
3	Imperial Valley	1979	Westmorland Fire	6.5	9.8	0.11	12.0
4	Coalinga	1983	Pleasant Valley P. P	6.4	8.4	0.30	39.4
5	Coalinga	1983	Oil City	5.2	9.5	0.37	13.6
6	Loma Prieta	1989	Corralitos	6.9	3.9	0.65	56.0
7	Loma Prieta	1989	Saratoga-Valley Coll	6.9	9.3	0.26	42.1
8	Landers	1992	Lucerne	7.3	2.2	0.73	133.4
9	Northridge	1994	Arleta-Nordhoff Fire	6.7	8.7	0.35	41.1
10	Northridge	1994	Sepulveda Hospital	6.7	8.4	0.75	77.7
11	Northridge	1994	Sylmar-Converter	6.7	5.2	0.85	121.0
12	Kobe, Japan	1995	KJMA	6.9	1	0.83	91.1
13	Kobe, Japan	1995	Port Island	6.9	3.3	0.35	90.7
14	Kobe, Japan	1995	Takatori	6.9	1.5	0.62	120.7
15	Chi-Chi	1999	CHY080	7.6	2.7	0.81	106.8
16	Chi-Chi	1999	CHY101	7.6	9.9	0.34	65.0
17	Chi-Chi	1999	TCU050	7.6	9.5	0.15	36.7
18	Chi-Chi	1999	TCU067	7.6	0.6	0.50	92.1
19	Northridge	1994	Newhall-Fire Station	6.7	9.4	0.11	7.0
20	Loma Prieta	1989	LG-Lexington	6.9	5	0.44	85.7

### Probabilistic seismic demand models

Through nonlinear time history analysis of 120 seismic waves in Sect.  5.3, the curvature of bridge piers was recorded, and the relationship between engineering demand parameter (EDP) and intensity measure (IM) underground motion was drawn according to the formula (3). In this study, the engineering demand parameter (EDP) is the curvature ductility of the pier bottom of the precast piers, and for the socket connecting piers, the curvature ductility of the precast piers at the top of the socket. The choice of PGV as IM to characterize the seismic intensity of selected ground motions has yielded multiple validations regarding its utility, efficiency, and proficiency for near-fault ground motions that may have pulse-like velocity effects^33,42^.

**Figure 16:**
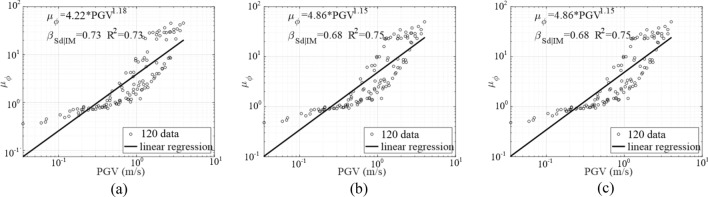
PSDMs for the curvature ductility demand. (**a**) CIP. (**b**) UC-1. (**c**) UC-2.

Figure 16 illustrates the established PSDMs for curvature ductility ${\mu }_{\varnothing }$ conditioned on PGV, where *R*^*2*^ is the correlation coefficient, and ${\beta }_{EDP|IM}$ is the dispersion of the fitting linear and quadratic curves, which is defined in Eq. (4). Because the *R*^*2*^ values (larger than 0.7) of each PSDM, it can be seen that the PSDMs have a good fit and can capture the relationship between a given PGV and the curvature ductility of piers.

### Component fragility curves

For a typical continuous girder bridge, the moment of the pier increases from the top to the bottom of the pier during an earthquake. This law also applies to the two piers discussed in this paper. For the pier with socket connection, the moment of the pier under an earthquake increases due to the increase in pier stiffness compared to a CIP pier. This increase in moment increases the risk of pier damage. However, the section of the pier at the socket connection increases, and the greater section stiffness makes the deformation of the shaft very small. In the 120 seismic waves calculated in this paper, the protective layer of concrete on the shaft doesn’t reach the peak strain and the outermost reinforcement doesn’t yield. The plastic hinge appears at the bottom of the cast-in-place pier, and the bottom of the socket-connected pier is almost undamaged. The curvature of the pier at the top of the socket is used for comparison as it is not meaningful to compare with the curvature at the bottom of the pier.

**Figure 17:**
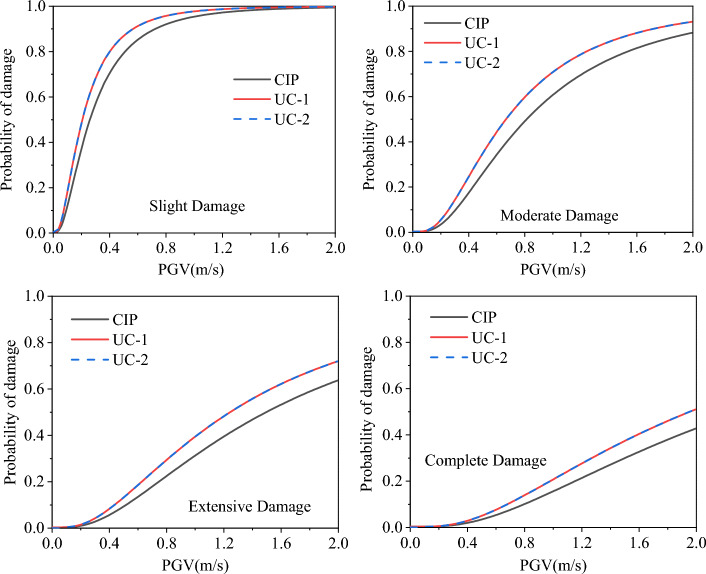
Seismic susceptibility curves of three specimens.

Figure 17 plots the fragility curves of the original bridge piers from minor to fully damaged states with CIP, UC-1, and UC-2, and it can be seen from the figure that the damage probabilities of the bridge piers connected by the cupped socket are increased under different damage states, which is similar to the analysis of the static test results in section "Experimental research". Table 7 extracts the damage probabilities of the three connection methods for different damage states at PGV at 1 m/s and 2 m/s. From the table, we can see that the fragility probability of the bridge pier with socket connection is 2.4%, 10.2%, 9.1%, and 5.1% higher than the damage probability of cast-in-place bridge pier at PGV of 1 m/s corresponding to four damage states in turn. This is due to the rise in the position of the plastic hinge, compared with the current pouring piers, the height of the socket connecting piers can be considered to be shortened, resulting in a higher probability of damage under the same seismic intensity.

**Table 7:** Damage probability of bridge piers under different damage states.

	Damage state	PGV(m/s)	CIP (%)	UC-1 (%)	UC-2 (%)
Probability of damage	Slight	1.0	95.4	97.8	97.8
		2.0	99.5	99.8	99.8
	Moderate	1.0	60.4	70.6	70.1
		2.0	88.2	93.1	93.1
	Extensive	1.0	31.0	39.1	39.2
		2.0	63.6	71.9	71.9
	Complete	1.0	15.3	20.4	20.4
		2.0	42.6	50.9	50.9

In contrast, there is no difference in the fragility to damage of piers with different materials of sockets, and the maximum difference in damage probability between UC-1 and UC-2 is only 0.1% for the same seismic strength and damage state as demonstrated in Table 7. This indicates that stronger socket restraint will no longer affect the fragility of bridge piers to damage under ground motions when the socket’s strength is sufficient to allow the plastic hinge position to rise above the socket. Therefore, in the following research, only the damage probabilities of UC-1 and CIP in earthquakes are compared.

### Measures to reduce seismic fragility of bridge piers with socket connections

Through the fragility analysis of the piers, it can be determined that the seismic performance of the piers connected by the socket is slightly lower than that of the cast-in-place piers under an earthquake. To have a better application of socket connection in engineering projects, this section discusses the design parameters of the socket connection and gives countermeasures to improve the seismic performance of socketed piers.

The most critical design dimension parameters of the socket are (1) the column embedment length-to-column diameter ratio, (2) the connection diameter-to-column diameter ratio, (3) the transverse reinforcement ratio in the connection region, and (4) the concrete strength of the connection. From the final effect, parameter (1) affects the stiffness of the bridge pier, and a higher connection depth will make the bridge pier above the socket bear more bending moments, which is not conducive to seismic resistance. So it is not recommended to increase the connection depth based on meeting the connection strength.

The parameters (2) (3) (4) ultimately affect the restraint capacity of the socket. To ensure that the plastic hinge appears on the pier, the socket should have sufficient restraint capacity, and through the results of the fragility analysis using different strengths of concrete in section "Component fragility curves", stronger lateral restraint does not improve the seismic performance of the bridge pier.

In addition to the dimensional parameters of the socket, the reinforcement ratio of the precast pier and the yield force of the adopted high-damping bearings can optimize the seismic performance of the piers with socket connections in terms of enhancing the capacity of the structure and reducing the response of earthquake to the piers, respectively, so the two parameters are analyzed in this section.

**Figure 18:**
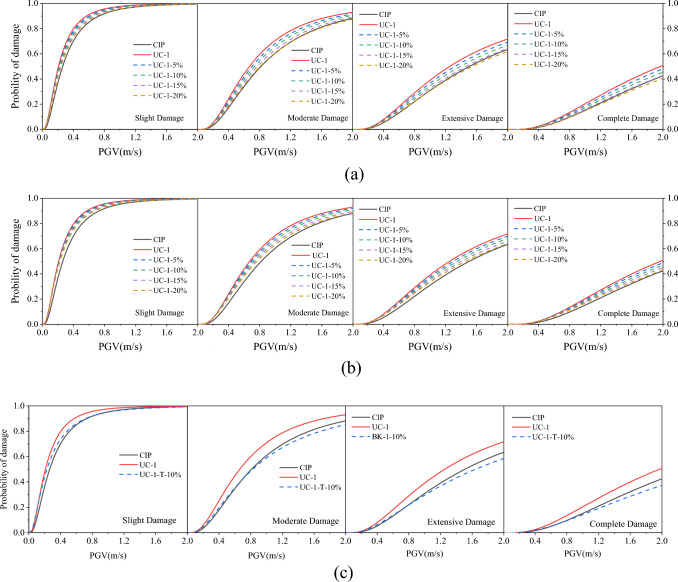
Fragility of piers using methods to develop seismic performance. (**a**) Increase of precast column’s reinforcement ratio. (**b**) Reduction of bearing yielding force. (**c**) Using the two methods (10% increase in reinforcement rate and 10% decrease in yield force of the bearing).

To evaluate the improvement of the precast pier reinforcement rate on the seismic performance of the piers, four models with the same details were developed with the actual bridge reinforcement rate increasing by 5%, 10%, 15%, and 20% in sequence. The analysis results are expressed as susceptibility curves, as shown in Fig. 18a. With the increase of reinforcement rate, the susceptibility of socket piers is decreasing, and with the increase of PGV, the damage probability is closer to that of cast-in-place piers. This is because when the earthquake intensity is low, the pier does not produce damage and the reinforcement does not play a full role, while during the strong earthquake effect, more reinforcement is involved in carrying the load. According to the analysis results, it is possible to obtain the same seismic capacity as cast-in-place piers in engineering practice by increasing the reinforcement rate of the pier by 15%, but this solution will increase the cost, so in the next subsection, the effect of the reduction of bearing yield force on the seismic performance of socket connected piers will be analyzed.

**Figure 19:**
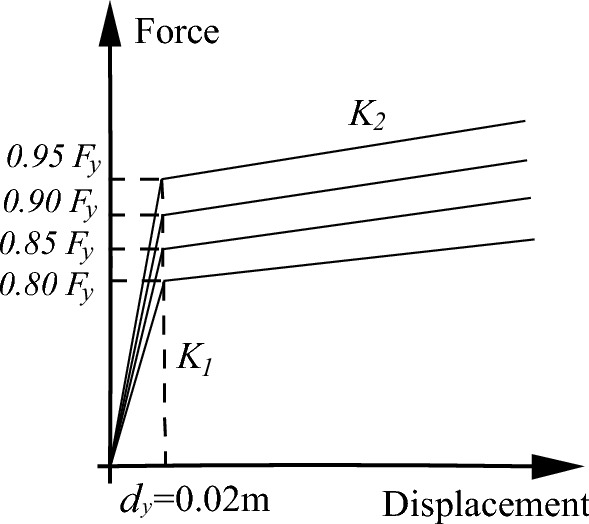
Force–displacement relationship of the bearing.

The reduction of bearing yield force will lead to an increase in bearing displacement and consume more energy, thus reducing the seismic response of the bridge pier. In this section, the bearing yield displacement is kept constant, the yield ratio is kept constant, and the bearing yield force is discounted by 5%, 10%, 15%, and 20% as shown in Fig. 19. Figure 18 (b) plots the susceptibility of bridge piers under different yield forces, with the reduction of bearing yield force, the susceptibility of bridge piers gradually approaches that of CIP piers.

Increasing the reinforcement ratio or reducing the bearing yield force can take the cupped socket piers to the same seismic performance as CIP piers respectively. However, the former requires an increase in cost and the latter leads to a larger structural displacement. Therefore, in actual engineering, it is optimal to use both methods in combination, such as a 10% increase in reinforcement ratio and a 10% reduction in bearing yield force, which will balance cost and structural displacement. Figure 18c illustrates that piers using the above method have a lower fragility to damage than CIP piers, thus using both increase of the reinforcement and reduction of the yield strength is a realistic method as an actual recommendation.

## Replacement

**Figure 20:**
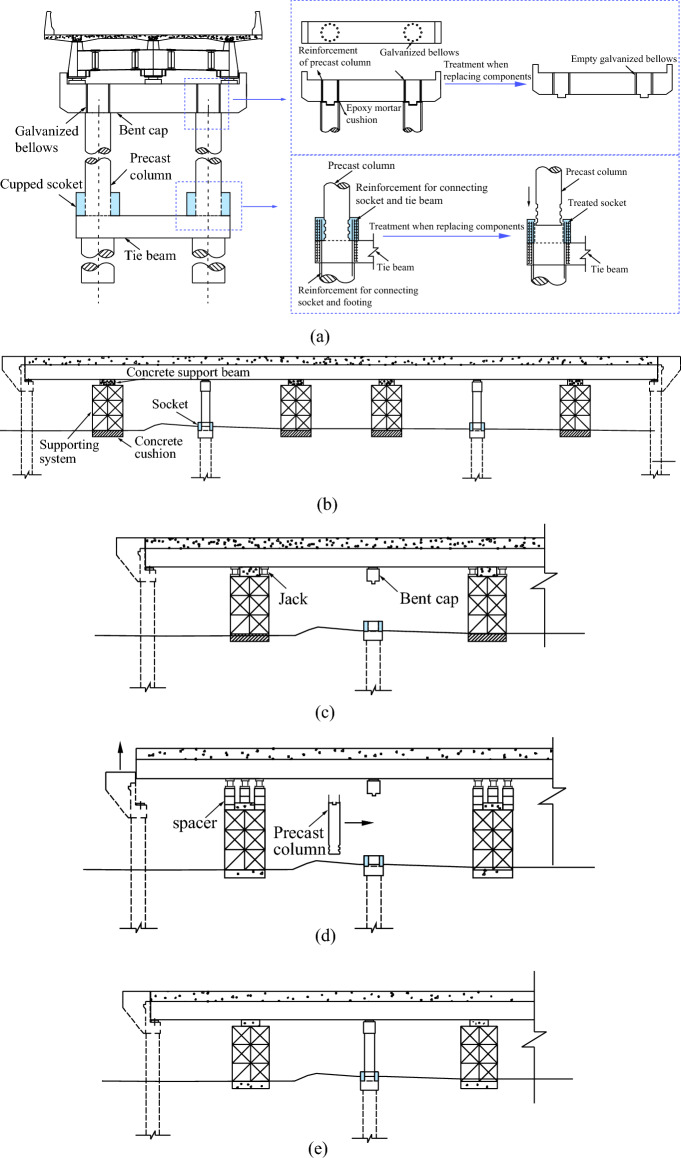
Diagram of bridge pier replacement. (**a**) Detail construction of bridge piers with cover beam and footing. (**b**) Erection of support system. (**c**) Chiseling of bridge piers and placement of jacks. (**d**) Beam jacking and installation of new piers. (**e**) Beam homing and maintenance of post-cast concrete.

This paper proposes an approach for connecting the piers and footings that allows for the damaged pier to be removed and replaced with a new precast pier after an earthquake. The replacement process involves four main steps, as shown in Fig. 20.

Step 1. To provide a foundation for the jacking operation, first lay a leveling layer on the ground. Then, erect a steel truss support system on the leveling layer, ensuring a certain distance between the support system and the beam. Finally, place concrete support beams to provide a foundation for the jacking operation.

Step 2. To address the resulting damage to the piers, the treatment of the cups is relatively simple. Only a cylindrical space with a diameter greater than that of the precast piers needs to be chiseled out. However, the treatment of the cover beam is relatively complex, the removal of piers should be left out of the protruding groove, the removal the grout, and reinforcement in the galvanized bellows should be cleaned up after the initial construction is restored to the situation. The jacks can be arranged simultaneously with the removal of the piers. They should be placed on both sides of the concrete support crossbeam.

Step 3. After completing the preparation work, start jacking. The jacking height should be the sum of the cup’s thickness and the cover beam’s height. Once the main jack reaches its maximum height, place the accompanying jack on the beam and jack it up. After the accompanying jack reaches the jacking height, restore the main jack and place a cushion underneath it. Then, jack up the main jack and repeat the process until the specified height is reached. The precast piers are then placed in the specified positions, and the girders are adjusted using jacks to ensure they are level.

Step 4. After completing the jacking process, grout the corrugated tubes of the bent cap socket and apply grout within the gap between the precast piers and the cupped socket. Once maintenance is finished, remove the support system and complete the repair.

## Conclusions

A new UHPC cupped socket connection for rapid replacement after earthquakes is proposed in this study, which satisfies the requirements of rapid bridge construction and post-earthquake recoverability. The seismic performance of this connection and CIP connection piers is compared by the quasi-static test, and the accuracy of the finite element models is verified. Combining experiments and numerical simulations, the following conclusions can be drawn.

1. The quasi-static test shows that the UHPC cupped socket connection has the same damage state as the CIP connection, and this connection can ensure the connection reliability of bridge piers and footings. The features of no damage to the UHPC socket and no bar connection through the piers and the footing are favorable for implementing the rapid replacement of bridge piers after the earthquakes.
2. Seismic fragility analysis shows that the material strength of the cupped socket at the design dimensions in this study has almost no effect on the seismic performance of the cupped socket connection. Therefore, with a sufficient construction period, common concrete can be used to pour the cupped socket instead of UHPC to reduce construction costs. However, it is unclear whether the use of common concrete can prevent damage to the sockets, and further research is needed.
3. The seismic fragility analysis shows that the fragility of piers with cupped socket connections is slightly higher than that of CIP piers. But the difference in the probability of damage under the four damage states is within 10.2%. Parametric analysis is conducted on the reinforcement rate of precast piers and the yield strength of bridge bearings using the cupped socket connection to ensure that both connections have the same seismic performance. The results show that compared with CIP columns, a 15% increase in reinforcement ratio or a 20% reduction in yield force of the bearings can achieve the same seismic performance of the inserted piers as CIP piers, and it is recommended to use both methods at the same time in actual engineering.

## Data Availability

The data supporting the findings of this study are available from the corresponding author upon request.
